# Vision–Language Models in Medical Imaging for Cancer Diagnosis: A Bibliometric Review

**DOI:** 10.3390/bioengineering13040466

**Published:** 2026-04-16

**Authors:** Musa Adamu Wakili, Aminu Bashir Suleiman, Kaloma Usman Majikumna, Harisu Abdullahi Shehu, Huseyin Kusetogullari, Md. Haidar Sharif

**Affiliations:** 1 Department of Artificial Intelligence, Abubakar Tafawa Balewa University, Bauchi 740272, Nigeria; musaadamuw@gmail.com; 2 Department of Cyber Security, Federal University Dutsin-Ma, Katsina 821101, Nigeria; 3 College of Intelligence and Computing, Tianjin University, Tianjin 300072, China; 4 School of Digital Engineering and Artificial Intelligence, Euromed University of Fes, Fes-Meknes 30030, Morocco; u.majikumna@ueuromed.org; 5 School of Engineering and Computer Science, Victoria University of Wellington, Wellington 6012, New Zealand; harisushehu@ecs.vuw.ac.nz; 6 Department of Computer Science, Blekinge Institute of Technology, 37141 Karlskrona, Sweden; huseyin.kusetogullari@bth.se; 7 Department of Computer Science, Capitol Technology University, Laurel, MD 20708, USA; hsharif@captechu.edu

**Keywords:** vision–language models, multimodal AI, medical imaging, bibliometric analysis, cancer diagnosis

## Abstract

The demand for advanced detection methods and accurate staging remains a global challenge in cancer diagnosis. Even though traditional deep learning models in medical imaging achieve high precision, they suffer from limited explainability and multimodal reasoning due to their black-box nature, thereby limiting their clinical applicability. To address this gap, recent research has increasingly explored multimodal approaches that integrate visual and textual clinical data to enhance diagnostic accuracy and interpretability. This study presents a bibliometric analysis of 408 publications from 2021 to 2025, collected from Web of Science and Scopus, using VOSviewer and R-Bibliometrix to map citation networks, co-authorship, and keyword co-occurrences. The results reveal a rapid growth from 1 publication in 2021 to 269 in 2025, with significant contributions from leading countries and institutions. Thematic analysis indicates a shift from conventional convolutional approaches toward transformer-based and self-supervised methods, alongside increasing attention to multimodal learning in cancer imaging tasks such as breast, lung, and brain cancer analysis. Overall, this study provides a structured overview of the evolving research landscape, highlighting key trends, emerging themes, and research gaps to inform future developments in multimodal artificial intelligence for cancer diagnosis.

## 1. Introduction

Cancer remains a leading cause of mortality worldwide, necessitating advanced detection methods and precise staging strategies to improve patient outcomes. While various malignancies present distinct clinical challenges for instance, the fatal outcomes associated with liver cancer especially hepatocellular carcinoma (HCC), the rising global incidence of colorectal tumors, or the high mortality rates of malignant melanoma they all share a critical requirement for early and accurate diagnosis. If adequate treatment is initiated early, survival rates improve dramatically, highlighting the urgent clinical need for robust, reproducible diagnostic auxiliary tools.

As stated by [1], addressing this global challenge requires improved strategies for early detection, accurate staging, and effective therapies. Malignant melanoma is among the deadliest forms of skin cancer, accounting for nearly 65% of skin cancer-related deaths, highlighting the need for accurate diagnostic methods. Early detection is crucial, as timely treatment can result in a 5-year survival rate exceeding 99% for certain skin lesions [2]. In colorectal cancer, malignant polyps are key precursors, and their early identification plays a vital role in prevention. Furthermore, early-stage cancers such as breast cancer often present with subtle clinical manifestations, stressing the importance of effective screening. Overall, this global burden highlights the urgent need to reduce cancer-related mortality in line with the broader objective of good health and well-being through improved prevention, early detection, and more effective treatment strategies.

The medical imaging has significantly advanced modern medicine by enabling non-invasive visualization for diagnosis, treatment planning, and follow-up [3,4]. Common imaging modalities, including X-ray, computed tomography (CT), magnetic resonance imaging (MRI), mammography, and colonoscopy, play critical roles in detecting and characterizing cancers such as breast, lung, colorectal, and pancreatic tumors [5,6,7,8]. In parallel, digital pathology, particularly through Whole Slide Images (WSIs), provides high-resolution tissue-level analysis and serves as a complementary modality for cancer diagnosis, staging, and prognosis, although it remains complex and time-consuming to interpret [9,10]. The integration of Artificial Intelligence (AI), especially in Computational Pathology (CPath) and deep learning applications, has further improved tasks such as cancer detection, subtyping, survival prediction, adenoma detection rates, real-time polyp histology assessment, and skin cancer classification [11,12,13]. Table 1 presents representative imaging modalities and AI methods commonly reported in the literature.

Traditional diagnostic methods, such as histopathological examination, can be invasive and time-consuming, and in some cases involve a degree of expert-dependent interpretation, which may affect efficiency and reproducibility in auxiliary decision-making [28]. Artificial Intelligence (AI) has significantly advanced diagnostic pathology and precision medicine by reducing subjectivity and enhancing image analysis through computer-aided diagnosis [29]. The shift from traditional methods to machine learning and deep learning has advanced computational pathology, driven by digital slide scanning, rapid AI research, large datasets, and high-performance computing [11]. To address these limitations, there has been significant interest in applying deep learning techniques to the analysis and recognition of digital pathology images [30]. The increasing demand for rapid, high-quality medical imaging, coupled with the rising complexity of diagnostic requirements, has created an urgent need for innovative computational solutions [3].

Machine learning and deep learning (DL), particularly CNN-based models, have achieved strong performance in image classification, anomaly detection, tissue segmentation, lesion detection, and disease diagnosis across X-ray, CT, MRI, breast cancer, skin lesions, and colonoscopy imaging [28,31,32,33]. However, AI development in medical imaging is constrained by limited annotated data and high labeling costs [11,34]. Emerging approaches such as self-supervised learning (SSL), transformer architectures, and diffusion models are helping address these challenges and improving complex pattern modeling in medical images [35,36]. Despite these advances, significant gaps remain in multimodal integration, interpretability, and alignment with clinical practice.

AI progress has not closed the gap in healthcare data processing, as most models remain single-modality despite clinicians working multimodally [37]. Current systems function as black boxes, lacking explainability, uncertainty estimates, and alignment with clinical standards [38]. Single-modality models limit diagnostic precision, and separating medical images from clinical text reduces personalization and accuracy [39]. Histopathology and other fields rely heavily on images, yet human reasoning depends on language found in reports and textbooks [13]. CNN-based deep learning models ignore key patient metadata and still depend on labor-intensive annotations [40]. Domain generalization remains weak due to limited use of structured clinical data, and image–text fusion is difficult. LLM use in surgery is minimal [41], and unstructured sensitive medical text raises privacy challenges requiring XAI [42]. Overall, the black-box nature and lack of interpretability of current AI models hinder their trustworthy deployment in clinical practice [43]. Recent studies highlight the role of LLMs in healthcare fact-checking and trustworthy AI. Despite their strong reasoning capabilities, they are prone to hallucinations, posing risks in clinical decision support. To mitigate this, approaches such as retrieval-augmented generation (RAG) and evidence-grounded reasoning have been proposed [44]. For example, scientific evidence-based fact-checking and graph-based retrieval frameworks like TrumorGPT improve factual reliability in LLM outputs [44,45]. These advances align with VLMs and MLLMs, where integrating multimodal data with reliable knowledge sources is key to building trustworthy medical AI systems.

**Figure 1:**
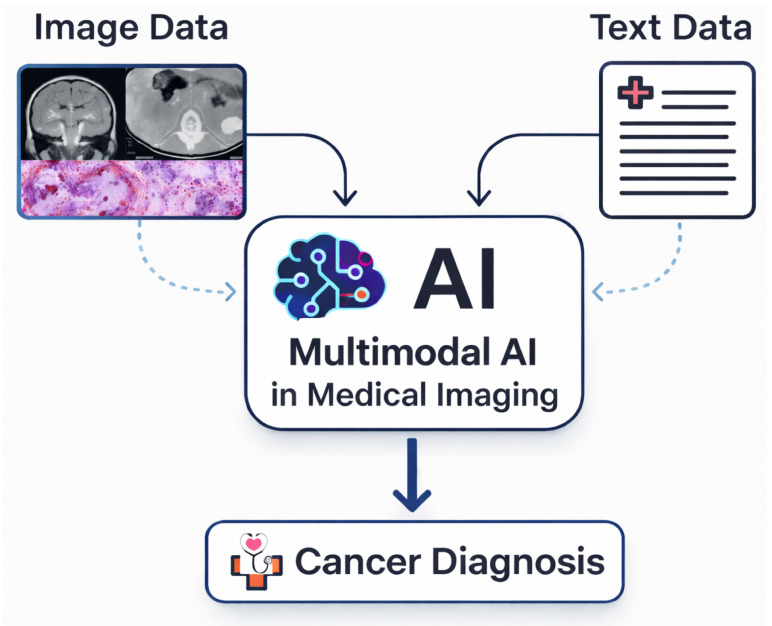
Integration of image data and clinical text for cancer diagnosis.

To contextualize the technological shift analyzed in this review, Figure 1 illustrates the foundational paradigm of Multimodal AI in oncology: the transition from isolated, single-modality image analysis to a unified architecture. By jointly processing visual data (e.g., MRI, histopathology) and clinical text (e.g., electronic health records, radiology reports), these models closely mimic human clinical reasoning, providing a conceptual baseline for the literature mapped in this study. The limitations of single-modality and text-only approaches have driven the development of multimodal AI models (Figure 1). Vision–Language Models (VLMs) and Multimodal Large Language Models (MLLMs) integrate visual and textual information, enabling more interpretable, explainable, and accurate medical image analysis [1,34,46]. VLMs, such as CLIP, SimVLM, and Radformer, align images and text through contrastive pretraining, supporting tasks like zero-shot anomaly detection, radiology report generation, and cancer image segmentation [5,47,48]. MLLMs, such as GPT-4V and Gemini-Vision, demonstrate strong few-shot and zero-shot learning, generalization, and integration of multimodal clinical data, addressing annotation scarcity and reducing physician workload [35,46,49,50]. However, these capabilities introduce important trade-offs between generalization and reliability. In high-stakes medical contexts, zero-shot and few-shot models may produce inaccurate or non-clinically grounded predictions when encountering unseen or complex cases, potentially compromising diagnostic safety. This highlights the need for rigorous validation, domain-specific adaptation, and human oversight to ensure trustworthy deployment. Applications of these models in healthcare, such as automated medical reporting and personalized treatment planning, illustrate their potential to enhance diagnostic accuracy, clinical workflow, and decision-making [51,52,53]. These developments emphasize the need for a bibliometric review to systematically map the current state of VLMs and MLLMs research in medical imaging for cancer diagnosis, identify key trends and influential studies, and inform future directions. The specific objectives of this bibliometric review are:

- To map the research landscape of Vision–Language Models (VLMs) and Multimodal Large Language Models (MLLMs) in medical imaging for cancer diagnosis, focusing on publication trends and growth.
- To identify influential contributions and collaboration patterns by analyzing key publications, authors, institutions, and countries using citation and productivity metrics.
- To analyze research themes and hotspots related to cancer types, imaging modalities, and clinical tasks.
- To identify emerging trends, research gaps, and future directions in multimodal AI applications for cancer diagnosis through bibliometric analysis.

By systematically analyzing the literature using bibliometric and science-mapping techniques, this study provides a comprehensive and structured foundation for researchers and clinicians, guiding future development toward robust, interpretable, and clinically trustworthy VLM solutions in computational oncology.

## 2. Materials and Methods

### 2.1. Data Sources and Retrieval Strategy

The data were extracted from two major scholarly repositories: Web of Science and Scopus, ensuring the comprehensiveness and representativeness of the dataset. Each of these databases, while extensive, had notable coverage limitations. Therefore, a multi-source search strategy was employed, a common approach in bibliometric research [54]. Web of Science was used as the primary source due to its authoritative standing in the field, particularly for engineering and computer science domains. It provides access to high-impact literature and includes a wide range of conference proceedings and journals that may not be fully indexed elsewhere. In addition to this, Scopus was incorporated to capture a broader scope of research across physical sciences, engineering, and life sciences, ensuring the inclusion of high-quality journal articles from Elsevier and other leading publishers. By combining these two databases, we were able to leverage Web of Science’s robust citation analysis tools and Scopus’s broader coverage to ensure the dataset’s completeness. This multi-source strategy is especially effective in bibliometric studies, offering a more thorough representation of the existing literature and its evolving trends [55]. While additional databases such as PubMed Central, IEEE Xplore, and arXiv are highly relevant to medical imaging and artificial intelligence research, they were not included as primary data sources in this study for methodological consistency and to avoid duplication bias. Web of Science and Scopus were selected because they provide high-quality indexing, standardized metadata, and robust citation tracking across both medical and engineering domains. PubMed primarily focuses on biomedical literature and has limited coverage of engineering conference proceedings, while IEEE Xplore is specialized toward technical publications and may not fully capture clinical studies. arXiv, although influential and identified in the citation analysis, primarily hosts preprints that may later appear in indexed journals, creating duplication and version control challenges. Therefore, Web of Science and Scopus were considered sufficient to provide a balanced and non-redundant representation of peer-reviewed and high-impact research. The presence of arXiv in citation results reflects its role as a dissemination platform rather than a primary curated data source.

Records for this bibliometric review were retrieved on 1 January 2026 using the specified search string. The constructed search string combines terms from three conceptual domains using the Boolean operators “AND” and “OR.” as shown in Table 2:

This study encompasses literature published between 1 January 2021, and 31 December 2025. A five-year timeframe was deliberately selected because the application of Vision–Language Models in medical imaging has experienced rapid, exponential growth during this specific period. Focusing the analysis on this window provides a highly relevant and concentrated map of state-of-the-art technological advancements and active research fronts within computational oncology.

### 2.2. Data Screening and Inclusion/Exclusion Criteria

Studies were included if the title and/or abstract indicated (i) a vision–language or image–text multimodal approach, (ii) an application in medical imaging, or other clinical images), and (iii) a clear oncology-related objective, such as cancer diagnosis, detection, classification, staging, grading, prognosis, biopsy support, screening, or early detection. Studies were excluded if they were outside the specified period, not in English, or not classified as an Article or Conference Paper, or if the title/abstract did not satisfy all three concept requirements (vision–language/multimodal and medical imaging and cancer-related clinical aim), including papers focused on non-oncology topics, non-medical images, text-only clinical analytics, or vision-only models without an explicit language–image component.

In total, 1974 records were retrieved, comprising 627 from Web of Science and 1347 from Scopus. After merging the datasets, 374 duplicate records were removed based on DOI, leaving 1600 unique records for screening. Next, records were screened using the title and abstract alongside the language and document-type filters. During this stage, 17 non-English records and 139 records with non-eligible document types were excluded. A further 1192 records were removed due to lack of relevance to the study topic based on title/abstract screening. Following these exclusions, 408 records remained and were included in the final bibliometric analysis.

Following duplicate removal, metadata harmonization was performed to ensure consistency across records. Variations in journal names like abbreviated vs. full titles, author affiliations, and keyword formats were standardized using a combination of automated string matching and manual verification. Institutional names were normalized to reduce fragmentation in affiliation analysis. Duplicate records were primarily identified using DOI matching. When DOIs were unavailable, title and author matching served as secondary criteria. The excluded document types (*n* = 139) included reviews, editorials, book chapters, letters, notes, and short communications, as these were not considered primary research contributions suitable for bibliometric analysis. Preprints were not explicitly included unless indexed within Web of Science or Scopus. When a preprint was subsequently published as a peer-reviewed article, only the indexed final version was retained to avoid duplication.

### 2.3. Bibliometric Analysis Techniques and Tools

The bibliometric indicators used in this study were calculated based on citation data obtained from Web of Science and Scopus at the time of data extraction. The h-index of a source is defined as the maximum number h such that the source has published h papers each receiving at least h citations. The g-index is defined as the largest number g such that the top g publications collectively received at least g^2^ citations, thereby giving more weight to highly cited articles. These indicators were computed using global citation counts as indexed in the databases, rather than being restricted solely to citations within the 408-document sample. To enable fair comparison across publications from different years, normalized total citations (Norm. TC) were computed by dividing the total citation count of each paper by the average citation count of all papers published in the same year. This approach mitigates temporal bias, where older publications tend to accumulate more citations than recent ones.

**Figure 2:**
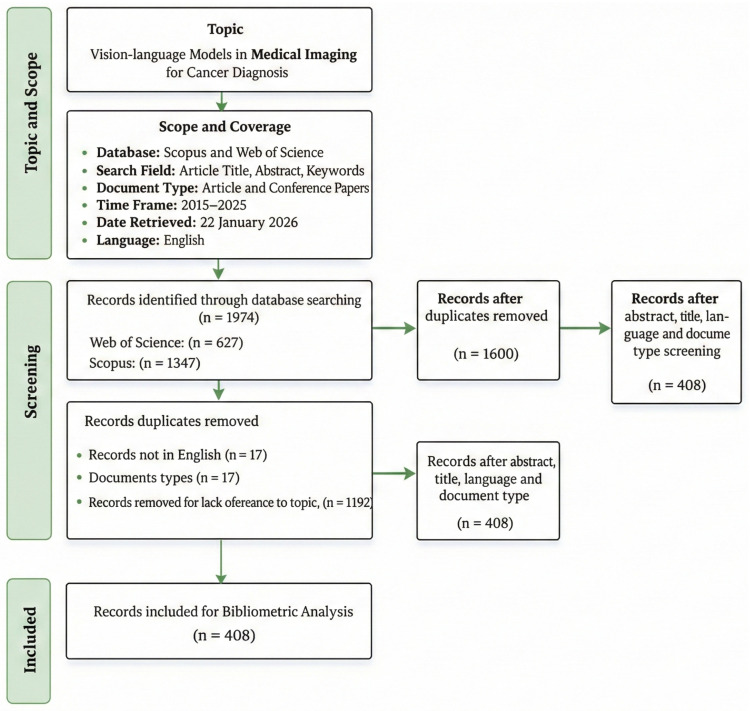
Bibliometric Analysis Workflow for VLM-in-Cancer-Imaging Research (2021–2025).

We conducted bibliometric analysis using a combination of descriptive indicators and network mapping. Descriptive statistics were used to summarize publication and citation trends, as well as the most productive authors, institutions, countries, and journals. Science mapping techniques were applied to generate co-authorship, co-citation, and keyword co-occurrence networks to identify collaboration patterns, influential knowledge bases, and major research themes. Network construction and visualization were performed using VOSviewer 1.6.20 [56] a widely utilized software tool specifically designed for constructing and visualizing bibliometric networks and R-Bibliometrix an R based application developed for bibliometric analysis [57] was used for data cleaning, summary statistics, and additional plots as shown in Figure 2.

Network construction and visualization were performed using VOSviewer [56] and R-Bibliometrix [57] was used for data cleaning, summary statistics, and additional plots as shown in Figure 2.

## 3. Results

### 3.1. Document Type

**Figure 3:**
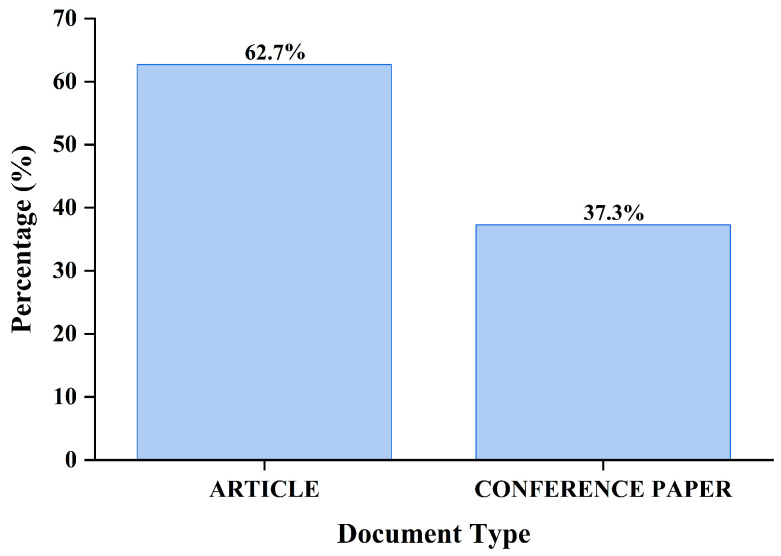
Document Type Distribution.

Figure 3 shows the distribution of the different document types in the dataset examined. The findings indicate that most documents are classified as “ARTICLE” (*n* = 256), accounting for about 62.7% of the total. This data shows that people in the field really like peer-reviewed journal articles. This is because they want to share their research findings through formal academic publishing channels. “CONFERENCE PAPER” ranks second among document types (*n* = 152), accounting for 37.3% of the total. The figure indicates that a significant amount of research is disseminated at academic and professional conferences, likely attributable to the field’s dynamic and technical nature, which benefits from prompt dissemination and collaborative discourse. The prominence of both document types highlights the dynamic interaction between journal-based and conference-based scholarly communication in this field.

### 3.2. Annual Scientific Production

**Figure 4:**
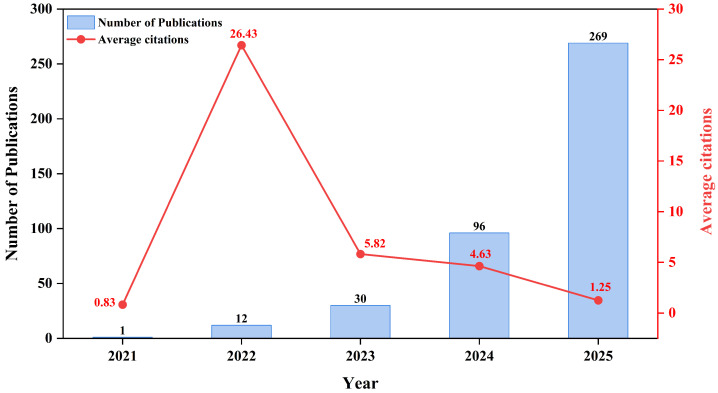
Exponential annual publication growth with baseline average per-publication citations.

The increase in the number of scientific papers published each year shows that research in the field is clearly on the rise from 2021 to 2025. As illustrated in the figure, the number of published articles began with 1 in 2021, followed by a slight increase to 12 in 2022. In 2023, there was a more noticeable rise, with 30 articles published. In 2024, the number of articles published continued to grow, reaching 96. The number of publications rose to 269 by 2025, showing that research output was growing at a faster rate. This steady, significant growth shows that more people are interested in using vision–language models for medical imaging to diagnose cancer. The rise is probably due to improvements in deep learning and artificial intelligence, as well as their growing use in medical imaging and the rise of multimodal techniques. The rapid growth in publications also shows that more people are working together, new technologies are being developed, and research funding is helping this field grow, as shown in Figure 4.

Although bibliometric growth is a sign of increasing scholarly interest, it is not a guarantee of clinical preparedness or architectural maturity. VLM architectures used in oncology differ, according to a more thorough technical analysis of the literature. Contrastive frameworks like CLIP, which are excellent at zero-shot classification by aligning images and text in a shared latent space but struggle with dense, localized reasoning necessary for tumor segmentation, were the mainstay of early foundational approaches. Models like BLIP (Bootstrapping Language–Image Pre-training) and LLaVA (Large Language-and-Vision Assistant) have become more popular in more recent, highly cited works. While LLaVA connects visual encoders with large language models to enable conversational AI capabilities for intricate, multi-turn clinical inquiries, BLIP introduces a multimodal mixture of encoder–decoder architectures, providing improved generation capabilities for radiology reports. The bibliometric transition from ‘image classification’ to ‘large language models’ (as seen in our thematic map) reflects this architectural evolution from discriminative contrastive models to generative, instruction-tuned MLLMs.

### 3.3. Most Relevant Sources

**Figure 5:**
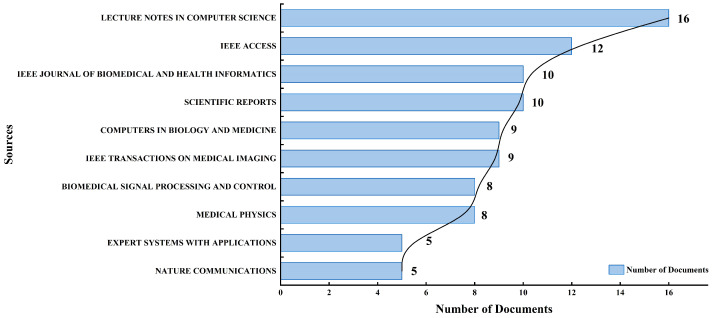
Leading journals ranked by number of publications.

Figure 5 shows the most important sources of publications that have helped the field, based on the number of articles that have been published. There are 16 articles in the Lecture Notes in Computer Science series, which demonstrate the importance of sharing conference-based research in the computational and applied sciences. IEEE Access comes next with 12 articles, underscoring its role as a multidisciplinary, open-access journal that helps new studies get published quickly. The IEEE Journal of Biomedical and Health Informatics and Scientific Reports each publish 10 articles, demonstrating that journals focused on interdisciplinary biomedical applications and open scientific communication are highly active. Computers in Biology and Medicine and IEEE Transactions on Medical Imaging are two other important sources, each with 9 articles. These demonstrate the importance of imaging and computational methods in the field. Biomedical Signal Processing and Control and Medical Physics each have eight articles, and Expert Systems with Applications and Nature Communications each have five articles. This distribution shows how the research area is multidisciplinary, connecting computer science, engineering, medicine, and health informatics through a variety of important publication platforms.

### 3.4. Most Cited Sources

**Figure 6:**
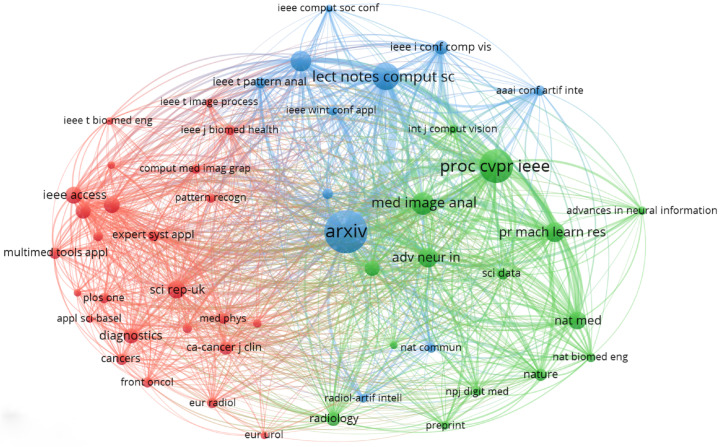
Most influential sources by total citation counts.

The most-cited publications in the dataset reveal the academic platforms that have been most important in shaping the research landscape. With 679 citations, arXiv stands out among them. This shows how important it is as a platform for quickly sharing preprints across fields such as medical imaging, computer vision, and machine learning. Blue nodes represent computer science and engineering-focused venues, green nodes denote medical imaging, machine learning, and clinical AI outlets, and red nodes correspond to general medical, applied science, and oncology journals. Following closely behind is the Proceedings of the IEEE Conference on Computer Vision and Pattern Recognition (CVPR), which has 404 citations. This shows how important it is as a place for cutting-edge research in visual computing. The Lecture Notes in Computer Science series has 275 citations, indicating that it remains useful for sharing information across many areas of computing. Medical Image Analysis (186 citations) and IEEE Transactions on Medical Imaging (155 citations) are two examples of journal-based sources that demonstrate the importance of peer-reviewed journals for advancing clinical and methodological research. Advanced Neural Information Processing Systems, Pattern Recognition, and Nature Medicine are also among the top sources, which shows how the field is appealing to people from many different fields and how deep the methods are. Figure 6 shows that this distribution includes conference proceedings, preprint servers, and high-impact journals. This shows that many different platforms help spread knowledge in both academic and practical fields.

### 3.5. Sources’ Local Impact

Table 3 presents the local impact of various publication sources within the field, assessed using bibliometric indicators such as the h-index, g-index, m-index, total citations (TC), number of publications (NP), and publication start year (PY). These metrics offer a comprehensive view of both the productivity and scholarly influence of the journals analyzed. Lecture Notes in Computer Science emerges as the most influential source, with the highest total citation count (1289) and the highest g-index (16), underscoring its role in publishing a substantial number of highly cited articles. IEEE Access also demonstrates strong performance, with 192 citations and an h-index of 6, highlighting its consistent visibility and relevance across the literature. Sources such as Biomedical Signal Processing and Control and Computers in Biology and Medicine maintain respectable h-index values of 6 and 5, respectively, indicating sustained impact. Meanwhile, Scientific Reports, IEEE Transactions on Medical Imaging, and Medical Physics show moderate scores across all metrics, reflecting a balanced mix of publication volume and citation performance. Interestingly, Nature Communications, despite having a low total citation count (27) and a g-index of just 3, records a high number of publications (58). This contrast may point to its recent inclusion in the dataset (beginning in 2022) and the natural delay in citation accumulation for newer articles. In summary, it indicates how journals like Lecture Notes in Computer Science and IEEE Access are central to the dissemination of impactful research in the field, while others represent emerging platforms that may gain influence over time.

### 3.6. Most Relevant Authors

The most prolific authors, based on the number of articles contributed and their fractionalized contributions, which account for co-authorship, are shown in Table 4. WANG J leads with 12 articles and a fractionalized score of 1.40, followed by LI Y with 10 articles and the highest fractional contribution of 1.46, indicating significant authorship roles. Other prominent contributors include ZHANG Y (9 articles), CHEN X and WANG X (8 articles each), and CHEN H and LIU Y (7 articles each). Despite similar article counts, differences in fractionalized scores reflect the extent of individual contribution, offering a more nuanced measure of author impact in collaborative publications.

### 3.7. Collaboration Network

**Figure 7:**
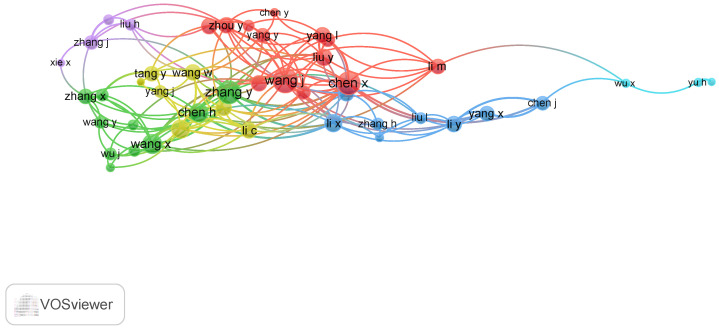
Authors Collaboration Network.

Figure 7 presents the authors’ collaboration network, offering a comprehensive visualization of the field’s co-authorship landscape. Each node represents an author, with node size proportional to their publication volume, and edges connecting them represent co-authorship links, with thicker lines indicating stronger collaboration. Red nodes form the largest, most central cluster, green and yellow nodes constitute a second major interconnected group, blue nodes represent a third distinct collaborative community, and purple and cyan nodes denote smaller, semi-peripheral or isolated research groups. Central and prominently sized nodes such as WANG J, ZHANG Y, WANG X, and LI Y reflect their high research productivity and extensive collaborative ties, placing them at the core of the network. Other notable contributors include CHEN X, LI X, LIU Y, CHEN H, ZHANG J, and YANG J, whose active participation forms tight-knit clusters, suggesting strong institutional or thematic partnerships. The presence of several color-coded clusters suggests distinct collaborative communities, potentially indicating specialized research subfields. Authors like CHEN S, TANG Y, YANG Y, XIE X, WU X, and LI H appear in semi-peripheral positions, possibly representing focused research groups or emerging networks. Additionally, relatively isolated nodes such as YU H and BAYER S suggest individual or niche contributions that, while impactful, are less integrated into the broader collaboration structure. Altogether, the network illustrates the dynamic and interconnected nature of scholarly activity in the field, highlighting both leading contributors and emerging voices shaping the research landscape.

### 3.8. Most Relevant Affiliation

**Figure 8:**
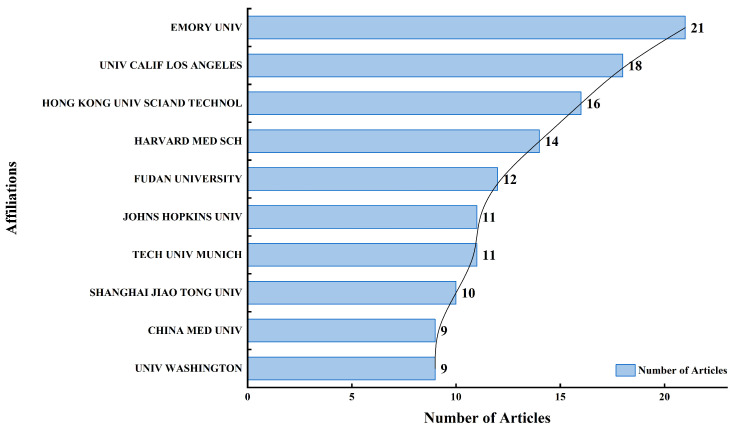
Leading academic institutions ranked by number of publications.

Figure 8 highlights the most active institutional contributors to the analyzed body of research. Emory University emerges as the leading affiliation, with 21 published articles, emphasizing its pivotal role in driving research in the field. University of California, Los Angeles (UCLA) follows closely with 18 publications, underscoring its strong research presence, particularly in biomedical and computational sciences. Hong Kong University of Science and Technology stands out with 16 contributions, reflecting its growing impact in interdisciplinary scientific domains. Harvard Medical School, a globally renowned institution, is also a major contributor with 14 articles, affirming its ongoing leadership in medical and clinical research. Other significant affiliations include Fudan University (12 articles), Johns Hopkins University, and Technical University of Munich (11 articles each), all of which are known for their strong academic reputations and emphasis on innovation. Shanghai Jiao Tong University, China Medical University, and the University of Washington each contributed 9–10 articles, showcasing international collaboration among Asia, Europe, and North America. Overall, the data reveal a diverse array of influential academic and research institutions actively shaping the landscape of scholarly output in the studied domain.

### 3.9. Most Global Cited Documents

Table 5 highlights the most influential publications in the field based on total citations and normalized impact. Earlier foundational works, such as Hatamizadeh A, 2022 [58], show high total citations, while more recent studies e.g., Lu 2024 and Yang 2022 [11,59] exhibit higher normalized citation scores, indicating strong and rapid influence despite their recency. Overall, the results reflect both long-term foundational contributions and emerging high-impact research driving current developments. To enable fair comparison across publications from different years, normalized total citations (Norm. TC) were computed by dividing the total citation count of each paper by the average citation count of all papers published in the same year. This approach mitigates temporal bias, where older publications tend to accumulate more citations than recent ones.

### 3.10. Most Cited Countries

**Figure 9:**
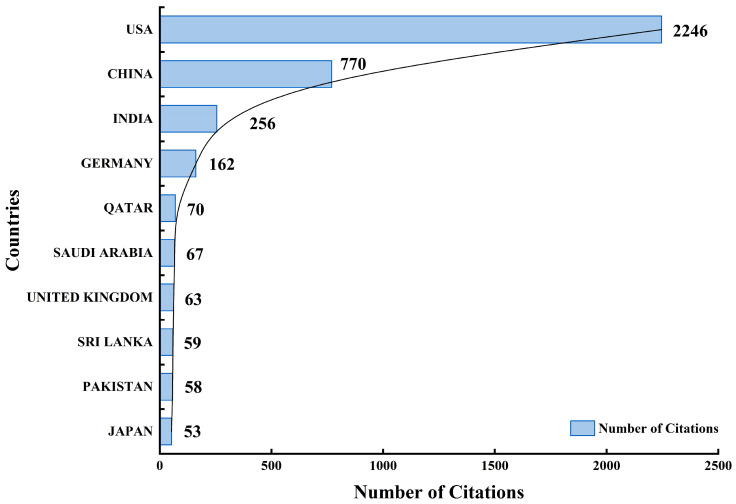
Cumulative citation impact for the most influential global contributors.

Table 6 and Figure 9 show that the USA dominates the field in both total citations and average citations per article, indicating strong research output and impact. While China and India contribute substantial volumes, their average citation rates are lower, suggesting broader but less concentrated impact. In contrast, countries such as Sri Lanka and Qatar achieve high average citations with fewer publications, reflecting selective yet highly influential research contributions.

### 3.11. Countries Scientific Production

**Figure 10:**
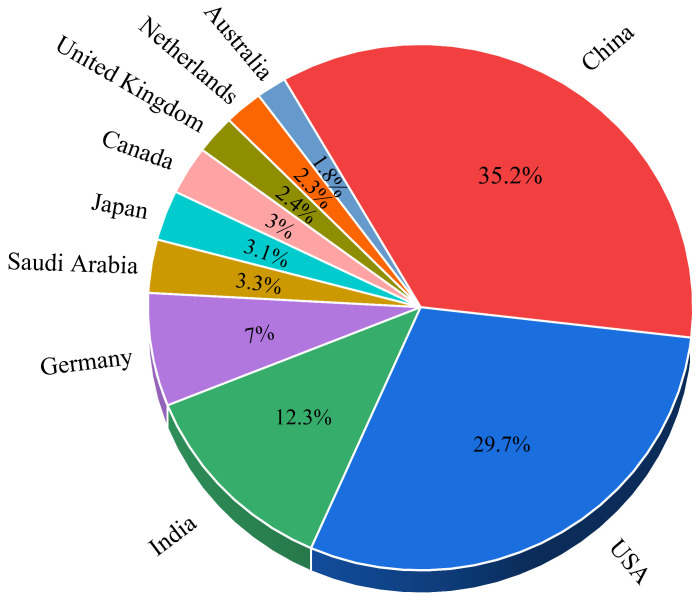
Percentage distribution of publication output across leading research countries.

Figure 10 illustrates that China and the USA lead global scientific production, reflecting their strong research capacity and sustained investment in the field. India and Germany follow with moderate output, while other countries contribute smaller but consistent publication volumes, indicating a broad and growing international research landscape.

### 3.12. Country Collaboration

**Figure 11:**
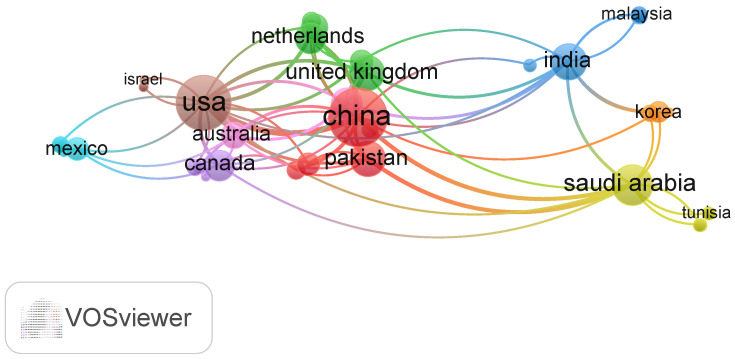
Internationalcollaboration network.

The country collaboration map in Figure 11 shows a globally interconnected research landscape dominated by strong partnerships between major scientific producers.red nodes form the central, high-output core cluster, green nodes represent the European collaborative community, blue nodes denote the Asian regional network, yellow/orange nodes constitute the Middle Eastern and South Asian collaborative group, and purple/cyan nodes correspond to North American, Oceanian, and Latin American research communities. The United States and China emerge as the central hubs, displaying the highest research output and the densest collaboration links, particularly with each other and with a wide range of partners across Europe and Asia. Several Asian countries, including India, Japan, and South Korea, form an active regional cluster anchored by China, reflecting growing research capacity in the region. European countries also appear well integrated, acting as bridges between the US- and China-centric networks through numerous intercontinental co-authorships. In contrast, Africa and much of South America show limited contributions, with lighter shading and fewer collaboration lines, indicating emerging or peripheral roles in the field. Overall, the map suggests a research ecosystem led by a US–China axis, supported by strong Asian and European secondary networks, and characterized by uneven global participation. Notably, while countries like Nigeria, South Africa, and other African nations contribute some research output in the field, they exhibit limited collaborative connectivity in the network, with sparse or absent co-authorship links to the global core.

### 3.13. Keyword Co-Occurrence

**Figure 12:**
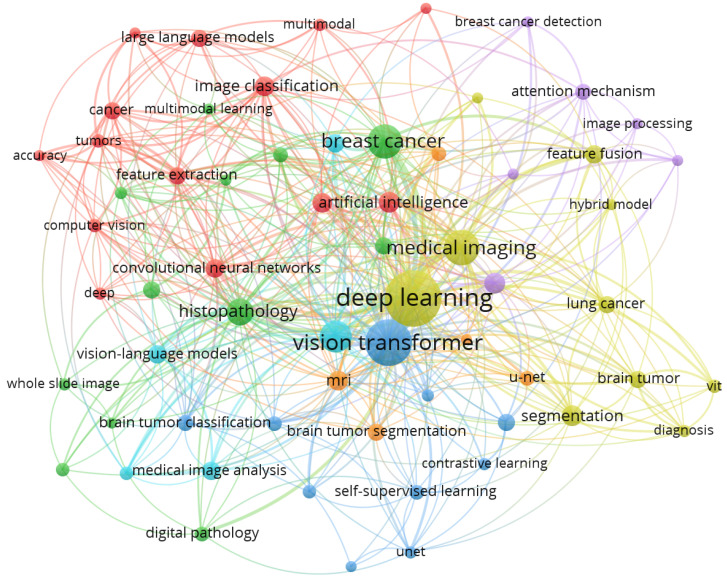
Keyword Co-Occurrence Network.

The keyword co-occurrence map Figure 12, generated using VOSviewer with a minimum occurrence threshold of five, resulted in a network of 124 keywords. For clarity, only those with a link strength above 10 are labeled. Red nodes represent the core computer vision and deep learning architecture cluster, green nodes denote the histopathology and digital pathology subfield, blue nodes correspond to self-supervised learning and vision transformer-based methods, yellow nodes form the medical imaging and clinical application cluster, and purple nodes represent the attention mechanism and feature fusion subfield. Within this network, the most frequent keywords “deep learning” (156 occurrences, total link strength 342), “medical imaging” (131, 289), and “vision transformer” (97, 241) form the central cluster. In terms of clinical applications, “breast cancer” appears 68 times (link strength 158), “lung cancer” 47 times (112), and “brain tumor segmentation” 39 times (98), confirming them as dominant research areas. Regarding architectural trends, “vision transformer” now surpasses “convolutional neural network” (52 occurrences, link strength 114) in both frequency and centrality. Finally, emerging terms such as “multimodal learning” (42 occurrences, link strength 96), “self-supervised learning” (38, 87), and “contrastive learning” (29, 71) appear as growing peripheral clusters, reflecting recent interest in annotation-efficient training strategies. The details keyword co-occurrence is depicted in Figure 12.

### 3.14. Thematic Map

**Figure 13:**
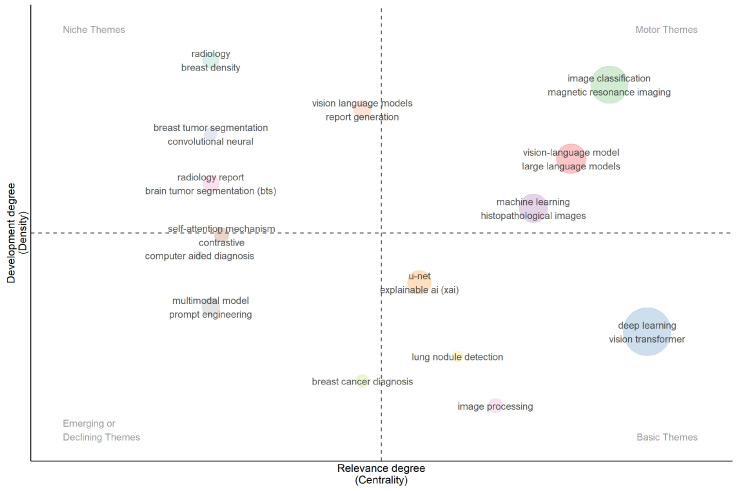
Thematic Map of VLM-in-Cancer-Imaging.

As shown in Figure 13, the thematic map positions research topics according to their development and relevance, resulting in four clear clusters that characterize the field. In the Motor Themes quadrant (high centrality, high density), above the horizontal divider and to the right of the vertical divider, there is a well-developed and highly connected cluster around “image classification” and “magnetic resonance imaging”, another between “vision–language model” and “large language models,” and a third between “machine learning” and “histopathological images”. In the Basic Themes quadrant (high centrality, lower density), “deep learning” and “vision transformer” make up a big group in the lower-right corner. This shows that they are a very relevant but not very dense foundation theme. “Image processing” is below the density divider and to the right of the centrality divider. “Lung nodule detection” is in the same lower-right half but closer to the central vert. In the Niche Themes quadrant (lower centrality, higher density), there are several specialized and internally cohesive topics on the left side above the horizontal divider. These include “radiology” with “breast density”, “breast tumor segmentation” with “convolutional neural”, and reporting/segmentation-oriented labels like “radiology report” and “brain tumor segmentation (BTS)”. These are mature sub-topics that are more isolated from the main topic. The map shows “multimodal model” and “prompt engineering” clearly below the horizontal line on the left. “Breast cancer diagnosis” is toward the bottom, close to the central vertical line (slightly on the left side). The centrality and density measures used in Figure 13 were calculated via R-Bibliometrix’s thematic map function, centrality (degree of connection between topics) is measured by the sum of link strengths to other themes, while density (internal cohesion) is measured by the average link strength within the theme. These metrics provide a quantitative basis for the quadrant classification. The underlying maturation process of the field is revealed through a critical interpretation of this thematic distribution. The Basic Themes quadrant’s placement of “vision transformer” and “deep learning” implies that these technologies are now the necessary infrastructure for contemporary medical imaging research rather than cutting-edge experimental tools. On the other hand, the Niche Themes quadrant’s isolation of “radiology reports” and “breast tumor segmentation” suggests that although these particular applications are highly developed, they are still isolated. Therefore, moving these dense, specialized niche applications into the Motor Themes quadrant and fully integrating them with the “vision–language models” and “large language models” that are currently generating the greatest interdisciplinary momentum will be crucial to the field’s future. Overall, Figure 13 demonstrates that the field is strongly anchored by foundational deep learning and transformer-based methods, while continuing to evolve through specialized imaging applications and new multimodal approaches.

## 4. Discussion

This analysis demonstrated an exponential growth in research output within the five-year time frame (2021–2025), indicating a definitive paradigm shift toward multimodal systems. Quantitatively, the field began with a single publication in 2021, followed by a steady rise to 12 in 2022 and 30 in 2023. By 2025, the output surged to 269 articles, representing a massive acceleration in scientific interest. This transition is formally documented by a preference for peer-reviewed journal articles, which account for 62.7% (*n* = 256) of the dataset, while conference papers account for 37.3% (*n* = 152). These metrics reflect a community-wide effort to emulate human clinical reasoning by integrating visual imaging with textual narratives, such as radiology reports and clinical histories.

The observed growth patterns and thematic shifts suggest that research on VLMs and MLLMs in medical imaging is rapidly evolving from early exploration toward broader adoption. The increasing focus on multimodal learning reflects a recognition of the limitations of single-modality approaches and the need for more comprehensive clinical reasoning. However, despite this rapid growth, the field remains in a developmental stage, with significant gaps in clinical validation, generalizability, and real-world deployment.

Through co-occurrence of keywords and thematic mapping, this study identifies deep learning, vision transformers, and medical imaging as the structural core of the field. While Convolutional Neural Networks (CNNs) have historically been foundational for anomaly identification, “motor themes” such as Vision Transformers (ViTs) and self-supervised learning have emerged as the dominant approaches. However, these trends are largely derived from bibliometric analyses and publication counts; the actual clinical efficacy, reproducibility, and robustness of these methods remain variable and underexplored. The bibliometric data support this conclusion via high citation counts for transformer-based works, such as the Proceedings of the IEEE Conference on Computer Vision and Pattern Recognition (404 citations) and foundational papers like Hatamizadeh et al. (2022) [58] with 1186 citations. These architectures excel at capturing complex spatial and linguistic relationships, yet their actual clinical efficacy remains variable as models move from “black box” predictions to explainable AI (XAI) outputs.

The scientific landscape is highly concentrated, defined by a dominant US–China axis. Quantitatively, China leads global production with 354 publications, while the USA follows with 299. However, the USA exhibits a higher research impact with 2246 total citations and an average of 35.1 citations per article, compared to China’s 770 total citations (8.4 average). Institutional leadership is anchored by Emory University (21 articles), UCLA (18 articles), and the Hong Kong University of Science and Technology (16 articles). While this reflects sustained investment in these regions, it also highlights a significant global disparity. Africa and large parts of South America show limited contributions, a gap that may be exacerbated by indexing biases in databases like Web of Science and Scopus, which often favor English-language journals.

Moreover, although multimodality poses a significant technical challenge, it is frequently presented in the literature as an intrinsic conceptual advantage. Systemically, it is computationally costly and architecturally challenging to align sparse, unstructured clinical text with high-dimensional, dense image data (like gigapixel Whole Slide Images). Modality imbalance is a common problem in models, where visual features are overshadowed by rich textual priors, resulting in hallucinations rather than evidence-based medical reasoning. Although models can correlate visual patterns with textual reports, they are still unable to deduce the underlying pathophysiological causality necessary for genuine clinical trust due to the lack of causal analysis in the existing literature.

Despite the promising research output, significant barriers to real-world deployment remain underexplored. The rationale for Vision–Language Models (VLMs) is built on overcoming the limitations of single-modality models, yet deployment is hindered by data privacy concerns, the high cost of annotated clinical data, and the unstructured nature of medical records. Moreover, current models often struggle to integrate high-dimensional sparse text with dense image data effectively. The bibliometric findings suggest that while research hotspots focus on breast cancer, lung nodules, and brain tumor segmentation, the field has yet to achieve universal robustness across heterogeneous patient populations and rare tumor types.

## 5. Limitations and Future Work

The reliance on Web of Science and Scopus for the period 2021 to 2025 may exclude relevant studies from other sources, and the rapid pace of AI advancements could render the findings outdated. The search terminology may also overlook pertinent papers, while restricting the corpus to English-language articles and conference papers limits broader representativeness. The analysis of growth is largely derived from bibliometric indicators and publication counts; however, the clinical efficacy, reproducibility, and robustness of these methods remain variable and insufficiently explored. Furthermore, the cataloging of AI applications does not account for real-world deployment challenges, including data constraints and integration into clinical workflows. Ultimately, this scientific landscape is constructed from publication and collaboration metrics, which may be influenced by indexing biases, language barriers, and the underrepresentation of certain regions in global databases. A fundamental limitation of bibliometric studies is that they measure research activity and citation impact, not clinical validity or technical reliability. High citation counts may reflect factors such as journal accessibility, author self-citation, or timely publication on a trending topic, rather than genuine scientific merit or clinical utility. Our analysis cannot assess the reproducibility, robustness, or safety of the underlying VLM methods, nor does it capture negative results or implementation failures. Consequently, the observed growth in publications should be interpreted as a sign of increasing academic interest, not as evidence of clinical readiness.

To address these limitations, future research should focus on:

- Quantitative benchmarking: Rather than relying solely on publication trends, the field would benefit from a more rigorous evaluation of AI methods through quantitative benchmarking. This involves the use of standardized performance metrics to facilitate objective comparisons, reproducibility studies to confirm the reliability of findings, and clinical trials to establish real-world effectiveness. Together, these approaches would provide a more comprehensive assessment of both technical validity and clinical value.
- Addressing data scarcity through synthetic data and LLM-assisted annotation: Data scarcity remains a major limitation in medical imaging, where obtaining annotated datasets is costly and requires expert knowledge. To address this, emerging approaches leverage synthetic data generation and LLM-assisted annotation to augment training data and reduce labeling effort. Techniques such as generative models, including variational autoencoders (VAEs) and diffusion models, enable the creation of realistic medical images, while multimodal LLMs can assist in generating structured annotations and reports. These strategies help mitigate class imbalance and improve model generalization. Similar approaches have demonstrated effectiveness in other domains, such as agriculture and cybersecurity, where VAE-based methods enhance performance under limited and imbalanced data conditions [67,68]. The wider development of generative AI, particularly diffusion models and text-to-image/video architectures, is crucial in overcoming these limitations, even beyond text-generating MLLMs. The development of multimodal rare-disease datasets and high-fidelity, synthetic tumor progression models is made possible by generative clinical simulation. These datasets can be used to train reliable diagnostic systems without jeopardizing patient privacy [69,70].
- Broader clinical integration: Effective translation of AI into practice requires investigating deployment strategies that extend beyond technical performance. Such strategies must account for heterogeneous patient populations to ensure generalizability and equity across diverse groups. They must also navigate complex regulatory frameworks to achieve approval and maintain compliance. Equally important is the seamless integration of AI tools into existing clinical workflows, ensuring they enhance efficiency and decision-making without adding cognitive burden or disrupting care delivery. Beyond oncology, generative AI is being incorporated into clinical practice, providing insightful insights from other specialized medical fields. For example, recent thorough surveys on generative AI models (2018–2024) in kidney care show how multimodal systems are effectively moving from theoretical frameworks to real-world applications in complex clinical decision support, medical education, and personalized patient management [71]. These domain-specific developments show that models that actively simulate clinical workflows and support dynamic treatment planning are necessary to overcome real-world deployment challenges.
- Developing Explainable and Interpretable Artificial Intelligence (XAI): Future research should focus on enhancing explainability in multimodal AI systems. Compared to traditional CNN-based approaches, which often provide limited visual explanations (e.g., heatmaps), VLMs and MLLMs offer improved interpretability by linking textual descriptions with specific image regions, enabling more transparent and clinically meaningful reasoning. However, current models still lack fine-grained grounding, where detailed textual outputs are explicitly aligned with localized pathological features. Developing such fine-grained vision–language alignment, along with standardized metrics for clinical faithfulness (e.g., adherence to BI-RADS or TNM staging), is essential for ensuring reliable decision support, regulatory acceptance, and trust in real-world clinical workflows.
- Combination of Multi-Institutional and Longitudinal Data: One of the major future opportunities is the transition to longitudinal multimodal reasoning rather than the snapshot diagnostics. The future models must use historical imaging data of the patient and emerging clinical histories to aid in monitoring of treatment and prognosis. Moreover, it requires studies on Federated Learning, or other more effective domain generalization methods that can enable models to learn on multi-institutional data without loss of patient privacy or centralized data storage.
- Inclusive global collaboration: To ensure that AI solutions benefit diverse populations, the field must actively encourage research partnerships with underrepresented regions. Such collaborations promote equitable access to AI technologies and help build capacity in settings that have historically been marginalized. They also contribute to the development of more diverse datasets, which in turn improve model generalizability and reduce algorithmic bias.

Additionally, the literature frequently asserts that VLMs improve workflow efficiency and accuracy, but these assertions are frequently not rigorously validated against established clinical benchmarks. Reporting reliable metrics like Area Under the Receiver Operating Characteristic (AUROC), sensitivity, and specificity in actual cancer detection situations must be a top priority for future research. Furthermore, the systemic problem of dataset bias where models trained on particular demographic or geographic datasets fail to achieve cross-institution generalization is largely ignored by current bibliometric trends. Significant regulatory restrictions are also brought about by the deployment of these multimodal models, such as FDA compliance for Software as a Medical Device (SaMD), data privacy (HIPAA/GDPR), and the legal ramifications of AI hallucinations in cancer diagnostics.

By addressing these points, the field can ensure that AI-supported clinical solutions are not only technologically advanced but also clinically effective, globally relevant, and ethically robust.

## 6. Conclusions

In conclusion, this bibliometric review highlights a critical period in the history of oncology research that will see the exponential increase in Vision–Language Models (VLMs) and Multimodal Large Language Models (MLLMs) between 2021 and 2025. This scientific output can be regarded as a sign of a wider understanding of the fact that the future of cancer diagnosis is in the union of computer vision and natural language processing. Through a combination of visual information with textual discourses, radiology reports and clinical histories, these highly developed AI structures are actually combating the natural constraints of the traditional, single-modality systems that tend to work in isolation. Replacement of black-box convolutional neural networks by more open-minded transformer-based models is beyond just technical improvement; it signifies a paradigm shift in the development of AI solutions that can be used to address the complex, multimodal reasoning of human clinicians. The paper manages to pinpoint that the correspondence between image and text representations, which is promoted by such a paradigm as Contrastive Language–Image Pre-training (CLIP), is defining a new basis of zero-shot and few-shot learning in computational pathology. This advancement is vital in the field given that it provides a practical substitute to the entirely prohibitively high and labor-intensive human annotation, especially in instances of rare tumor type or large-scale digital pathology scans. Based on the benefits of using free-text descriptions as supervision signals, these models are not only improving the accuracy of diagnosis and generalizability but they are also able to deliver the interpretability required to gain the trust of both medical practitioners and patients.

The United States and China dominate both productivity and collaboration networks, while institutional leadership is concentrated among a small group of universities. These findings provide a structured mapping of research trends, influential contributions, and emerging topics to inform future work in this field.

## Figures and Tables

**Table 1:** Modalities and methods across cancer types from CNNs to VLMs.

Cancer Type	Imaging Modality	AI Method
Breast Cancer [14,15]	Mammography, MRI,	CNN, Transformer, VLM
Colorectal Cancer [16,17]	Colonoscopy	CNN, DL, VLM
Skin Cancer [18,19,20]	Dermoscopy	CNN, DL, MLLM
Lung Cancer [21,22]	Chest X-ray, CT	CNN, Transformer, VLM
Pancreatic Cancer [23,24]	CT Scan, ERCP, MRI,	DL, Transformer
Prostate Cancer [17,25]	MRI, Histopathology	CNN, VLM, Foundation Models
Brain Tumor [26,27]	MRI	CNN, Transformer

**Table 2:** Categorized Boolean terms for VLMs, imaging, and cancer.

Category	Keywords
Vision–Language Models	“vision-language model ” OR “VLM” OR “vision language model ” OR “multimodal foundation model ” OR “vision-language foundation model” OR “CLIP” OR “Contrastive Language-Image Pre-training” OR “visual language model” OR “image-text model” OR “multimodal deep learning” OR “multimodal AI” OR “multimodal artificial intelligence” OR “vision transformer” OR “ViT” OR “language” OR “LLaVA” OR “BLIP” OR “Flamingo” OR “medical multimodal”
Medical Imaging Terms	“medical imaging” OR “radiology” OR “radiograph” OR “X-ray” OR “CT” OR “computed tomography” OR “MRI” OR “magnetic resonance imaging” OR “ultrasound” OR “sonography” OR “histopathology” OR “pathology” OR “whole slide imaging” OR “WSI” OR “mammography” OR “PET” OR “positron emission tomography” OR “dermatoscopy” OR “endoscopy” OR “colonoscopy” OR “medical image” OR “clinical image”
Cancer-related Terms	“cancer” OR “neoplasm” OR “tumor” OR “tumour” OR “malignan” OR “oncology” OR “carcinoma” OR “adenocarcinoma” OR “sarcoma” OR “lymphoma” OR “leukemia” OR “leukaemia” OR “diagnosis” OR “detection” OR “classification” OR “staging” OR “grading” OR “prognosis” OR “biopsy” OR “screening” OR “early detection”

**Table 3:** Local Bibliometric Impact of Top Publication Sources.

Source	h_Index	g_Index	m_Index	TC	NP	PY
Biomedical Signal Processing and Control	6	8	2.00	82	8	2024
IEEE Access	6	12	1.50	192	12	2023
Lecture Notes in Computer Science	6	16	1.20	1289	16	2022
Computers in Biology and Medicine	5	8	1.25	80	9	2023
IEEE Journal of Biomedical and Health Informatics	5	8	1.00	79	10	2022
Scientific Reports	4	8	1.33	65	10	2024
IEEE Transactions on Medical Imaging	3	7	0.75	58	9	2023
Medical Physics	3	5	1.00	72	5	2024
Nature Communications	3	3	0.60	27	58	2022

**Table 4:** Prolific authors ranked by publication volume and fractionalized impact.

Authors	Articles	Articles Fractionalized
Wang J	12	1.40
Li Y	10	1.46
Zhang Y	9	1.19
Chen X	8	0.95
Wang X	8	1.04
Chen H	7	1.04
Liu Y	7	0.90
Li X	6	0.95
Liu X	6	0.74
Wang Y	6	0.82

**Table 5:** Top 10 publications ranked by total and normalized citations.

Paper	DOI	TC	TC/Year	Norm. TC
Hatamizadeh A, 2022 [58]	https://doi.org/10.1007/978-3-031-08999-2_22	1186	237.2	8.97
Lu My, 2024 [11]	https://doi.org/10.1038/s41591-024-02856-4	316	105.33	22.77
Xu H, 2024 [60]	https://doi.org/10.1038/s41586-024-07441-w	100	66.67	11.77
Yang J, 2022 [59]	https://doi.org/10.1016/j.csbj.2021.12.028	388	194.00	31.97
Tummalas S, 2022 [61]	https://doi.org/10.3390/curroncol29100590	132	26.4	1.33
Wu C, 2023 [62]	https://doi.org/10.1109/ICCV51070.2023.01954	78	19.5	3.5
Lu My, 2023 [63]	https://doi.org/10.1109/CVPR52729.2023.01893	196	196.0	16.33
Steyaert S, 2023 [64]	https://doi.org/10.1038/s43856-023-00276-y	15	7.5	0.41
Wu H, 2024 [65]	https://doi.org/10.1109/TASE.2023.3292373	59	19.67	4.25
Gamage L, 2024 [66]	https://doi.org/10.3390/electronics13040680	54	18	3.89

**Table 6:** Top 10 countries ranked by citation volume and average impact.

Country	TC	Average Article Citations
USA	2246	35.1
China	770	8.4
India	256	4.8
Germany	162	10.8
Qatar	70	17.5
Saudi Arabia	67	8.4
United Kingdom	63	9.0
Sri Lanka	59	29.5
Pakistan	58	8.3
Japan	53	5.3

## Data Availability

The data presented in this study are available on request from the corresponding author.

## Abbreviations

The following abbreviations are used in this manuscript:

AI	Artificial Intelligence
VLMs	Vision–Language Models
XAI	Explainable AI
SSL	Self-supervised Learning
Cpath	Computational Pathology
LLM	Large Language Model
MRI	Magnetic Resonance Imaging
WSI	Whole Slide Imaging
ViT	Vision Transformer
CLIP	Contrastive Language–Image Pre-training
ERCP	Endoscopic Retrograde Cholangiopancreatography
MLLMs	Multimodal Large Language Models
DL	Deep Learning
WSIs	Whole Slide Images
BC	Breast Cancer
HCC	Hepatocellular Carcinoma
CRC	Colorectal Cancer
